# Development of a gRNA Expression and Processing Platform for Efficient CRISPR-Cas9-Based Gene Editing and Gene Silencing in Candida tropicalis

**DOI:** 10.1128/spectrum.00059-22

**Published:** 2022-05-11

**Authors:** Yujie Li, Lihua Zhang, Haiquan Yang, Yuanyuan Xia, Liming Liu, Xianzhong Chen, Wei Shen

**Affiliations:** a Key Laboratory of Industrial Biotechnology, Ministry of Education, Jiangnan University, Wuxi, Jiangsu, China; b School of Biotechnology, Jiangnan University, Wuxi, Jiangsu, China; c State Key Laboratory of Food Science and Technology, Jiangnan University, Wuxi, Jiangsu, China; Hubei University of Medicine

**Keywords:** *Candida tropicalis*, CRISPR interference system, RNA Pol III promoter, genome editing, gRNA expression element

## Abstract

Candida tropicalis, a nonmodel diploid microbe, has been applied in industry as a chassis cell. Metabolic engineering of C. tropicalis is challenging due to a lack of gene editing and regulation tools. Here, we report a tRNA:guide RNA (gRNA) platform for boosting gene editing and silencing efficiency in C. tropicalis. As the endogenous tRNA-processing system enables autocleavage for producing a large number of mature gRNAs, a tRNA^Gly^ sequence from the genome of C. tropicalis ATCC 20336 was selected for constructing the tRNA:gRNA platform. In the CRISPR-Cas9 system, the tRNA:gRNA platform proved to be efficient in single-gene and multi-gene editing. Furthermore, based on the tRNA:gRNA platform, a CRISPR interference (CRISPRi) system was developed to construct an efficient dCas9-mediated gene expression regulation system for C. tropicalis. The CRISPRi system was employed to regulate the expression of the exogenous gene *GFP3* (green fluorescent protein) and the endogenous gene *ADE2* (phosphoribosylaminoimidazole carboxylase). Different regions of *GFP3* and *ADE2* were targeted with the gRNAs processed by the tRNA^Gly^, and the transcription levels of *GFP3* and *ADE2* were successfully downregulated to 23.9% ± 4.1% and 38.0% ± 7.4%, respectively. The effects of the target regions on gene regulation were also investigated. Additionally, the regulation system was applied to silence *ERG9* (squalene synthase) to enhance β-carotene biosynthesis in a metabolically modified C. tropicalis strain. The results suggest that the endogenous tRNA^Gly^ and the CRISPRi system have great potential for metabolic engineering of C. tropicalis.

**IMPORTANCE** In the nonmodel yeast Candida tropicalis, a lack of available RNA polymerase type III (Pol III) promoters hindered the development of guide RNA (gRNA) expression platforms for the establishment of CRISPR-Cas-mediated genome editing and silencing strategies. Here, a tRNA:gRNA platform was constructed. We show that this platform allows efficient and precise expression and processing of different gRNAs from a single polycistronic gene capable of mediating multi-gene editing in combination with CRISPR-Cas9. Furthermore, in combination with dCas9, the tRNA:gRNA platform was efficiently used for silencing of exogenous and endogenous genes, representing the first CRISPR interference tool (CRISPRi) in C. tropicalis. Importantly, the established CRISPRi-tRNA:gRNA tool was also used for metabolic engineering by regulating β-carotene biosynthesis in C. tropicalis. The results suggest that the tRNA:gRNA platform and the CRISPRi system will further advance the application of the CRISPR-Cas-based editing and CRISPRi systems for metabolic engineering in C. tropicalis.

## INTRODUCTION

Candida tropicalis is an industrial microorganism with a special ω-oxidation pathway that is applied in the transformation of *n*-alkanes to aliphatic long-chain *α*, *ω*-dicarboxylic acids (DCs) (1–3). In recent years, C. tropicalis has been used in the production of xylitol (4–6) and polyhydroxybutyrate (PHB) (7) and bioremediation of heavy metals in environmental protection (8). C. tropicalis has many advantages, such as robust tolerance to furfural in the pretreatment of lignocellulosic biomass (8) and the availability of inexpensive, raw, starchy agro-products in PHB production (7).

Clustered regularly interspaced short palindromic repeats (CRISPR) system is a prokaryotic immune system that operates via CRISPR-associated (Cas) proteins that can recognize specific nucleic acids with guidance of a CRISPR RNA (crRNA). Some of those Cas proteins, especially CRISPR-Cas9, are being harnessed as tools for genome editing (9). Recently, a CRISPR interference (CRISPRi) has been established as a general tool for specific regulation of gene expression in both prokaryotes (10–12) and eukaryotes (9). The CRISPRi system consists of catalytically inactive Cas9 protein (dCas9) and a chimeric guide RNA (gRNA) (11, 12). Guided by the gRNA, the dCas9 protein binds to the target DNA region via Watson-Crick base pairing (11). If the binding site is within the promoter region or the coding sequence of a gene, RNA polymerase binding or elongation will be sterically blocked to some extent, resulting in transcriptional repression of the target gene and decreased protein production.

The CRISPRi system provides a tool to manipulate host genomes without irreversibly knocking out genes. Moreover, it has been utilized for rewiring and regulating metabolic networks in microbial cell factories. This regulation system can modulate biosynthesis pathway fluxes to balance mevalonate (MVA) pathway genes in terpenoid production (13, 14). In yeast cells, CRISPR regulation systems have been developed in Saccharomyces cerevisiae (15–17), Schizosaccharomyces pombe (18), Pichia pastoris (19), and Yarrowia lipolytica (20). However, a CRISPRi system has not been constructed in C. tropicalis, hindering the regulatable manipulation of gene expression in this organism.

When applying CRISPR tools, both RNA polymerase type II (Pol II) and RNA polymerase type III (Pol III) promoters can be used to produce gRNAs. Transcription from RNA Pol III promoters is different from that from RNA Pol II promoters responsible for mRNAs. RNAs transcribed by RNA Pol III remain in the nucleus instead of being transported to the cytoplasm. Among them, U3 and U6 are early RNA Pol III promoters commonly used to produce gRNAs (21). However, the functions of many RNA Pol III promoters are not yet clear in many species, and their applications are very limited. Researchers have paid attention to the central adapter molecules, tRNAs, which are highly expressed in cells, accounting for 4 to 10% of total cellular RNAs (22). In the maturation process of tRNAs in organisms, tRNAs undergo ribozyme cleavage at specific sites. Therefore, a tRNA:gRNA platform contributes to precise and efficient gRNA processing with desired 5′ targeting sequences. Compared with other gRNA expression techniques, the advantages of the tRNA:gRNA platform are as follows. (i) The tRNA:gRNA platform enables broad-host range applications, because the tRNA-processing system exists in almost all organisms (23). It fills the gap in available RNA Pol III promoters in new hosts (24). (ii) The tiny size is more suitable in producing different gRNAs from a single polycistronic gene (25). The length of nuclear encoded eukaryotic and prokaryotic tRNAs is less than 90 nucleotides (26). Accordingly, it leaves more space for vectors with limited packaging capacity (27). (iii) It hardly causes potential cell burden. In some gRNA techniques, heterologous endonuclease Csy4 or ribozymes are needed (28), which carries additional risk of toxicity in heterologous gene expression (25). In rice plants, the tRNA:gRNA platform was first used in multiplex genome editing and chromosomal-fragment deletion. This showed that tRNAs could produce large amounts of gRNA (25). Further, the tRNA-gRNA platform was applied in a S. cerevisiae multiplexed engineering system (GTR-CRISPR). The 2-promoter GTR-CRISPR allowed simultaneous disruptions of 8 genes with 87% efficiency (29).

In the present work, we developed a gRNA expression strategy and a CRISPRi system in C. tropicalis. We first obtained an endogenous tRNA encoding gene (tRNA^Gly^) from C. tropicalis ATCC 20336. It could serve as an available RNA Pol III promoter for expression of multiple gRNAs mediating single-gene disruption. Additionally, a tRNA:gRNA platform expressing multiple gRNAs for Cas9-mediated targeting was designed. A dCas9-based CRISPRi system was designed, which, in combination with the tRNA:gRNA platform, was applied to regulate exogenous and endogenous gene transcription. Regions downstream and upstream of the initiation codon were chosen, and suitable target sites in both regions were used to downregulate transcription of respective genes. The repression ability of the CRISPRi system in C. tropicalis was further verified in regulating β-carotene biosynthesis in C. tropicalis.

## RESULTS

### Analysis of endogenous tRNAs in C. tropicalis ATCC 20336.

**FIG 1:**
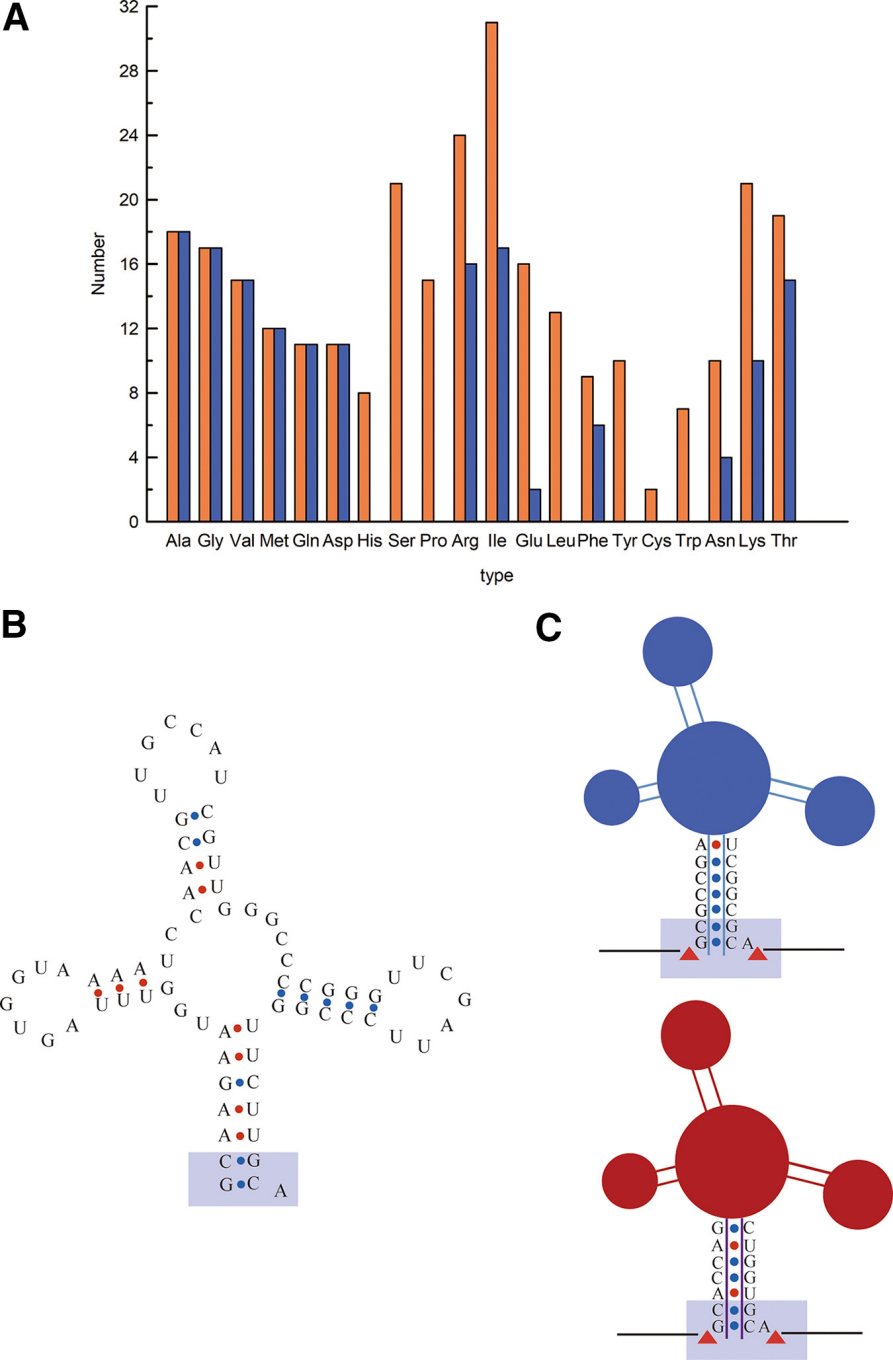
Information on endogenous tRNAs in C. tropicalis ATCC 20336. (A) Statistical result of tRNAs in the genome of C. tropicalis ATCC 20336. Orange refers to the total quantity. Blue refers to the number of tRNAs without introns. (B) The secondary structure of a tRNA^Gly^ sequence in C. tropicalis ATCC 20336. (C) Based on references 23 and 26, a partial sequence diagram of previously reported tRNA^Gly^ in Y. lipolytica (blue) and the one in rice (purple). The yellow square frame highlights the consistent nucleotides.

Utilizing tRNAscan-SE software (30), the whole genome of C. tropicalis was searched for tRNA-coding sequences. After excluding eight pseudogenes, we obtained 20 types of tRNAs related to transporting different amino acids in different quantities. In addition, among the various tRNAs, some were predicted to contain introns in the coding sequence (Fig. 1A). The six types of tRNAs predicted without introns were tRNA^Ala^, tRNA^Gly^, tRNA^Val^, tRNA^Met^, tRNA^Gln^, and tRNA^Asp^. Since the tRNA^Gly^ sequences lack introns and are short in length, they are suitable as RNA Pol III promoters. Therefore, we aligned the 17 sequences of the endogenous tRNA^Gly^ in C. tropicalis ATCC 20336, together with publicly available tRNA sequences of C. tropicalis MYA 304 and C. tropicalis 121 (see Fig. S1 in the supplemental material). Interestingly, we found that some of the C. tropicalis tRNA^Gly^ sequences were highly similar. Since we aimed to develop a tRNA able to serve as a potential promoter for transcription and processing of multiple gRNAs from one array, the secondary structure was also analyzed with tRNAscan-SE (30). Comparing the C. tropicalis tRNA^Gly^ (Fig. 1B) with the previously reported tRNA^Gly^ in Y. lipolytica (31) and rice (25), even though the sequences are quite different, the nucleotides close to the ribozyme cleavage sites are the same (Fig. 1C). As the ribozyme cleavage sites of the tRNA^Gly^ sequences were confirmed in other studies, we concluded that the ribozyme cleavage sites were consistent in the coding sequences of tRNA^Gly^.

### Functional analysis of the tRNA^Gly^ as an available RNA Pol III promoter.

**FIG 2:**
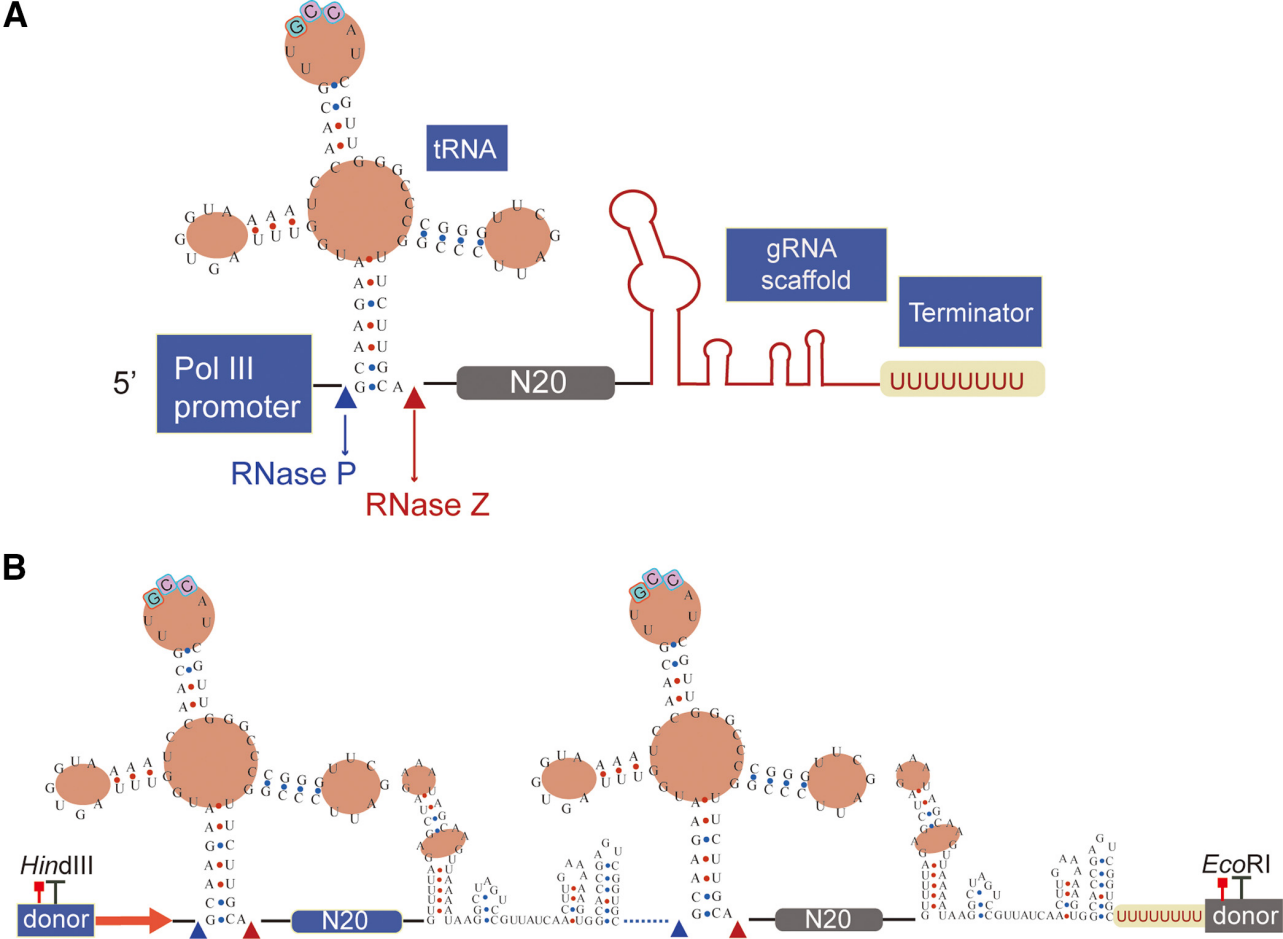
The tRNA:gRNA platform with the tRNA^Gly^ sequence. N20 refers to 20-nt targeting sequence in gRNAs. (A) gRNA for single gene target. (B) gRNA for double-gene targets.

**FIG 3:**
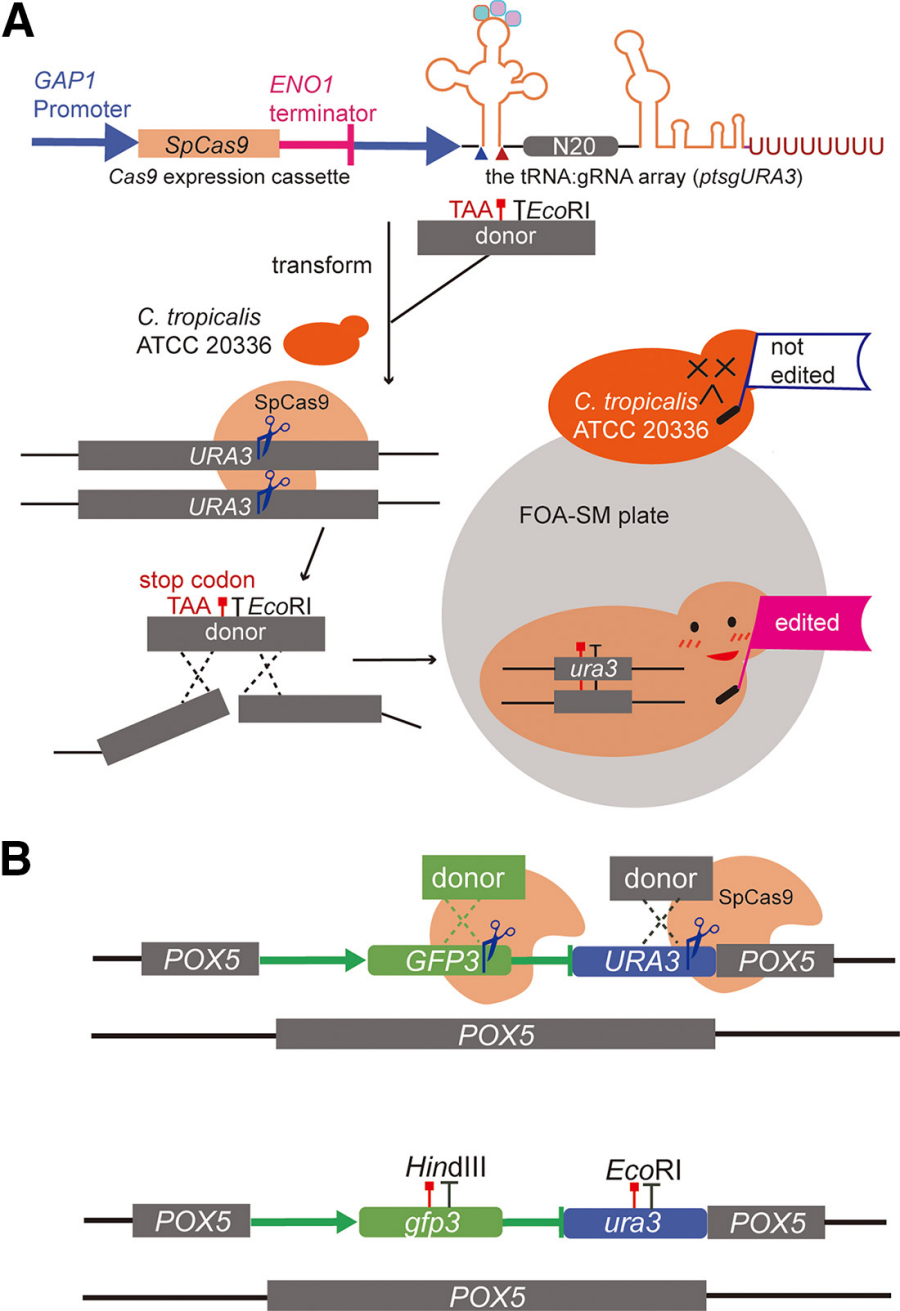
Single-gene and multi-gene knockout with a reconstructive CRISPR-Cas9 system. (A) The transient *Cas9-ptsgURA3* fragment used in two copies of *URA3* knockout. *SpCas9*, codon optimized *Cas9* gene from Streptococcus pyogenes; N20 refers to 20-nt targeting sequence in *URA3*. FOA-SM plate, SM supplemented with 2 g/L (wt/vol) 5-fluoroorotic acid. (B) Multi-gene knockout with the aid of the tRNA:gRNA platform.

To verify the availability of the candidate tRNA^Gly^, we fused a transient CRISPR-Cas9 cassette with the tRNA:gRNA array targeting *URA3* (*ptsgURA3*) (Fig. 2A, Fig. 3A, and Sequence S1). The final *Cas9*-*ptsgURA3* construct and the donor were transformed into C. tropicalis ATCC 20336. The donor was designed to carry an EcoRI restriction site and contained 50-bp homologous regions upstream and downstream of *URA3*. One hundred transformants on the screening plate were verified by PCR and EcoRI digestion. For this, the genomic DNA of the colonies was isolated and used as the template in PCR using the primers URA3-F and URA3-R (Table S2). The results of EcoRI digestion and sequencing showed that *URA3* was successfully edited in all colonies in three independent experiments.

### Multi-gene disruption was achieved with high efficiency using the tRNA:gRNA platform.

After demonstrating adequate efficiency for single-gene editing, the tRNA:gRNA platform was tested for multi-gene editing. We constructed the tRNA:gRNA platform targeting *GFP3* and *URA3*, paying particular attention to the junction of the gRNAs (Fig. 2B, Fig. 3B). More precisely, the polyU tail in the gRNA scaffold was removed between the consecutive gRNAs, based on an effective design for tRNA-gRNA direct fusions for gene editing in Y. lipolytica (31). The transformants on the screening plate were verified by PCR using primers URA3-F, URA3-R, GFP3-F, and GFP3-R listed in Table S2. EcoRI digestion, HindIII digestion, and DNA sequencing analysis were performed for assessing multi-gene editing efficiency (Fig. S2). At first, transformation of the two donors and the tRNA:gRNA platform reached only a 5% knockout efficiency for both genes. The donors were then fused to the termini of the tRNA:gRNA platform to enhance the editing rate (Fig. 2B), and 71.0 ± 4.8% of C. tropicalis GU colonies were correctly edited in the genome (Table 1).

**TABLE 1:** Disruption efficiency of multi-genes using the CRISPR-Cas9^*a*^^,^^*e*^ system with an RNA polymerase type III promoter

gRNA and donor	No. of colonies selected from FOA-SM plates	No. of correct recombinants	Homologous recombination efficiency^*b*^ (%)
Transient^*c*^	100, 110, 120	5, 5, 5	4.6 ± 0.4
Transient DNA fragment^*d*^	105, 108, 120	69, 78, 90	71.0 ± 4.8

a *Cas9* was integrated in the genome of host yeasts.

b The percentage was based on PCR analysis and enzyme validation of *ura3* and *gfp3* using genomic DNA from colonies isolated from FOA-SM plates.

c gRNA and the donors were transformed as independent DNA fragments.

d The gRNA array targeting *URA3* and *GFP3* flanked with donors.

e Results are means ± standard deviation.

### The CRISPRi system effectively represses the exogenous gene *GFP3*.

To implement the CRISPRi system in C. tropicalis, a synthetic Cas9 gene, based on the Cas9 gene from Streptococcus pyogenes, was inactivated by amino acid point mutations (*dSpCas9*, N10A, and H840A) (11). The 3′ terminus of *dSpCas9* was appended with a triple repeat of the SV40 nuclear localization signal, and the expression cassette consisted of the strong constitutive *GAP1* promoter and the *ENO1* terminator (32). The expression cassette was transformed into C. tropicalis Cu-206 to generate C. tropicalis D1. Since it was verified that the *GAP1* promoter supported high expression of genes in our previous work (32), we further transformed the reporter *GFP3* cassette with a *GAP1* promoter in the present work. The resulting strain was named C. tropicalis *GFP3* interference 0 (Gi0), and *dSpCas9* expression hardly affected the growth of C. tropicalis (Fig. S3).

**FIG 4:**
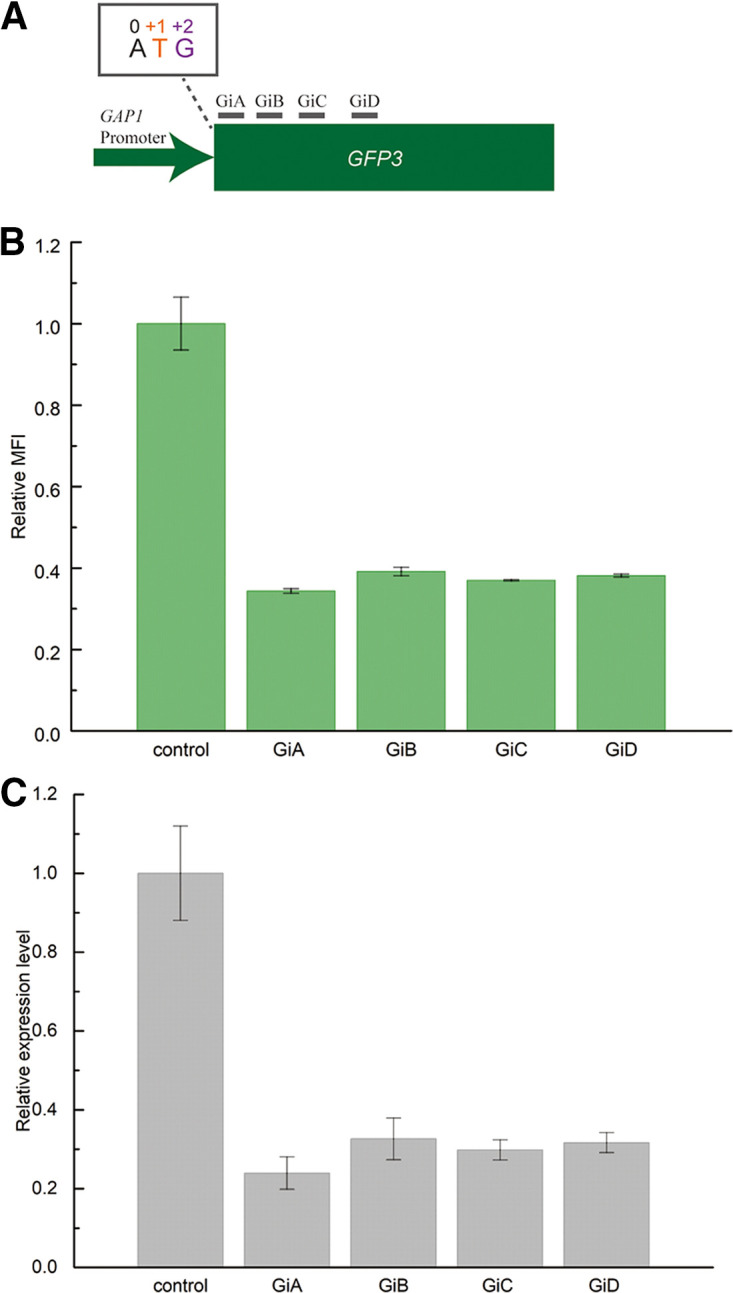
Regulation of *GFP3* expression by targeting the CDS region. (A) Different regions for gRNA design for targeting *GFP3*. (B) Relative mean fluorescence intensity (MFI) of the yeasts integrated with different cassettes to regulate *GFP3* expression. (C) Relative transcription levels of *GFP3* in the yeasts. Data are represented as mean ± standard deviation from at least three biological replicates.

**FIG 5:**
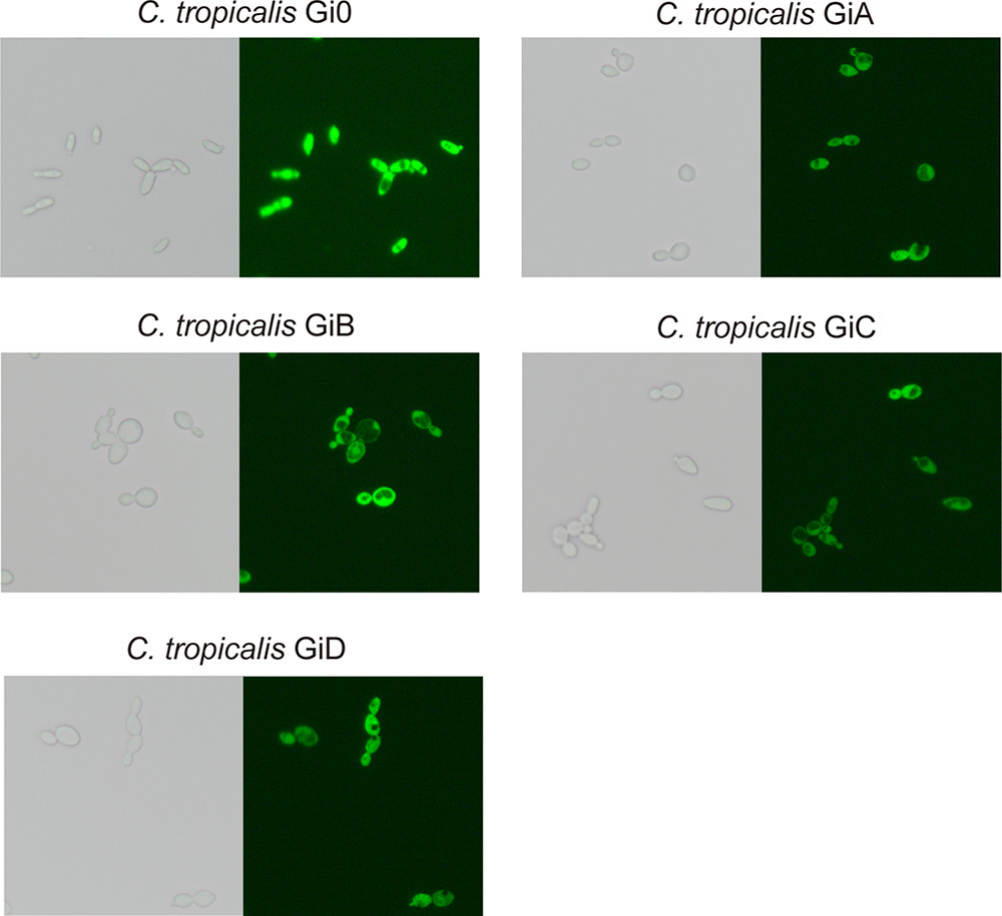
Fluorescence observation of C. tropicalis. The cells under the brightfield (left) and the excitation light (right). The parameters of the imaging were kept consistent. C. tropicalis Gi0 was the control.

Since the expression of both *dSpCas9* and *GFP3* depend on the strong constitutive *GAP1* promoter, the designed targeting region should be within the coding sequence (CDS) instead of the promoter sequence of *GFP3* to avoid disturbing the expression of *dSpCas9*. Four different regions in the CDS of *GFP3* were selected, based on which complementary gRNAs were generated (Fig. 4A): GiA (+6), GiB (+48), GiC (+96), and GiD (+249). The first nucleotide of 5′-N20 targeting sequences in *GFP3* (Table. S1) was marked to show the distance from the translation initiation codon (ATG) (Fig. 4A). After integrating the *GFP3* interference (Gi) cassette into the *POX5* locus in the genome, the green fluorescence intensity of the control (C. tropicalis Gi0) was stronger than that of the others, as observed by fluorescence microscopy (Fig. 5).

To gain an insight into the exact fluorescence of the mutants, the mean fluorescence intensity (MFI) of the single yeast cell was determined by flow-cytometry analysis. The relative MFI values of C. tropicalis GiA, C. tropicalis GiB, C. tropicalis GiC, and C. tropicalis GiD were 34.3 ± 0.5%, 39.1 ± 1.0%, 37.0 ± 0.1%, and 38.1 ± 0.4%, respectively. The relative expression levels of *GFP3* were measured further (Fig. 4B). The *GFP3* expression level in C. tropicalis GiA was repressed to 23.9% ± 4.1%, and in the other strains, the values were similar (Fig. 4C). Thus, it was confirmed that the CRISPRi system in C. tropicalis could repress the expression of exogenous genes. Selection of the CDS region proved reliable for achieving relatively high suppression.

### Interference effects differ among different gRNAs.

**FIG 6:**
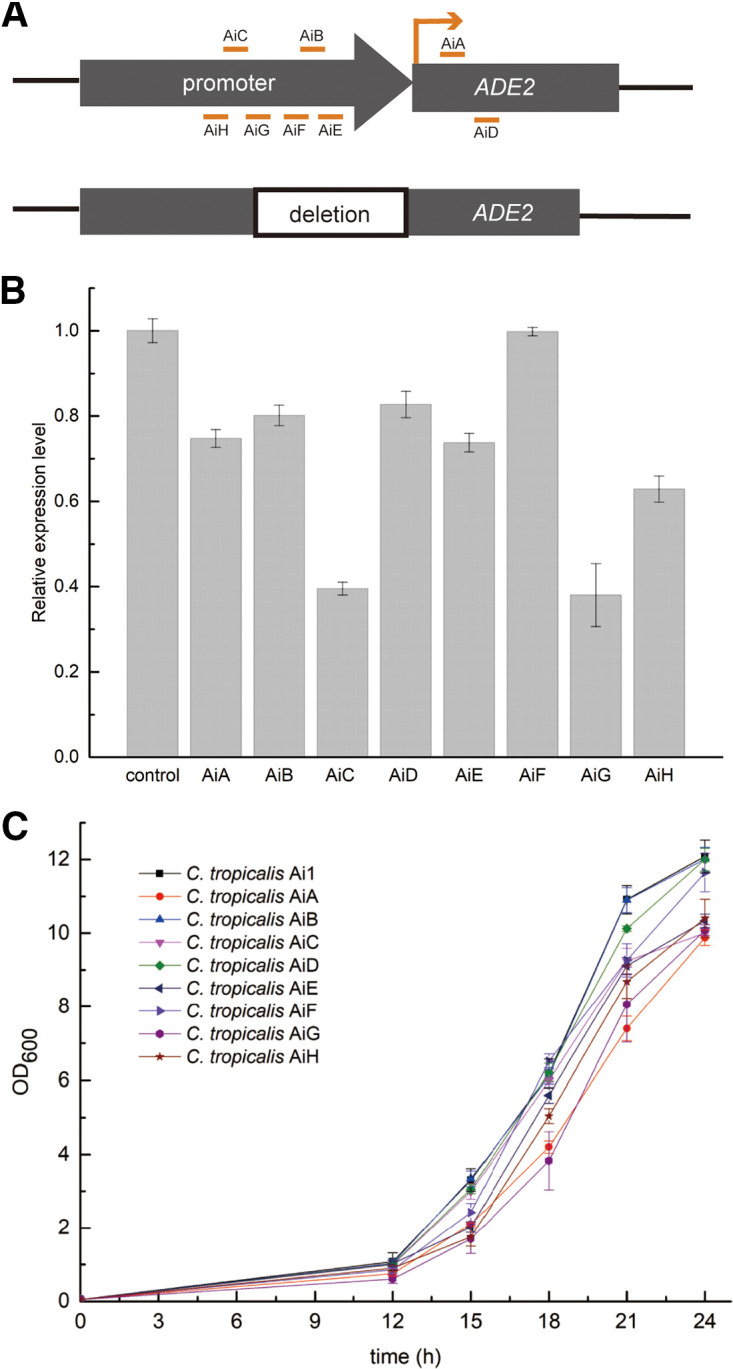
Regulate *ADE2* expression by targeting the promoter region. (A) Different regions for N20 design in *ADE2*. (B) Relative transcription levels of *ADE2* in the yeasts. Data are represented as mean ± standard deviation from at least three independent occasions. (C) The growth curve of the C. tropicalis mutants in MM. The data represent the means ± standard deviations of biological triplicates.

We investigated whether the targeting sites upstream and downstream of the translation initiation codon (ATG) were both available for regulating the target genes. *ADE2*, an important gene in the adenine biosynthesis pathway, was selected as the endogenous reporter gene. The promoter and the coding region of the endogenous genes in diploid yeast differed to various extents. We first knocked out one copy of *ADE2* in C. tropicalis D1, and the other copy was maintained, generating the C. tropicalis strain Da. Eight different regions in *ADE2* were chosen to design gRNAs (Fig. 6A): AiA (+164), AiB (−119), AiC (−212), AiD (+286), AiE (−19), AiF (−150), AiG (−195), and AiH (−345). The detailed targeting sequences are listed in Table S1. Quantitative real-time PCR (qRT-PCR) was used for the measurement of downregulation of *ADE2* (Fig. 6B). The regions AiC and AiG achieved obvious downregulation, with relative expression levels of 39.5 ± 1.5% and 38.0 ± 7.4%, respectively. However, in C. tropicalis AiF, there was no obvious downregulation of *ADE2*. For the other targeted regions, the relative expression levels ranged from 62.8 ± 3.0% (AiH) to 82.7 ± 3.1% (AiD). Thus, the promoter region in C. tropicalis is suitable for flexible regulation in the CRISPRi system.

To further analyze whether adenine biosynthesis was affected, the cell density (optical density at 600 nm [OD_600_]) of C. tropicalis mutants in which *ADE2* was downregulated was measured every 3 h. The growth curves of C. tropicalis mutants showed that silencing of *ADE2* resulted in growth suppression (Fig. 6C). The growth rate was in accord with the *ADE2* expression level, and C. tropicalis AiG grew slowest in the first 12 h among the nine strains. On the MM plate, the colony of C. tropicalis AiG appeared smaller than that of C. tropicalis Ai1 (Fig. S4).

### Applying the system to metabolic regulation of β-carotene.

**FIG 7:**
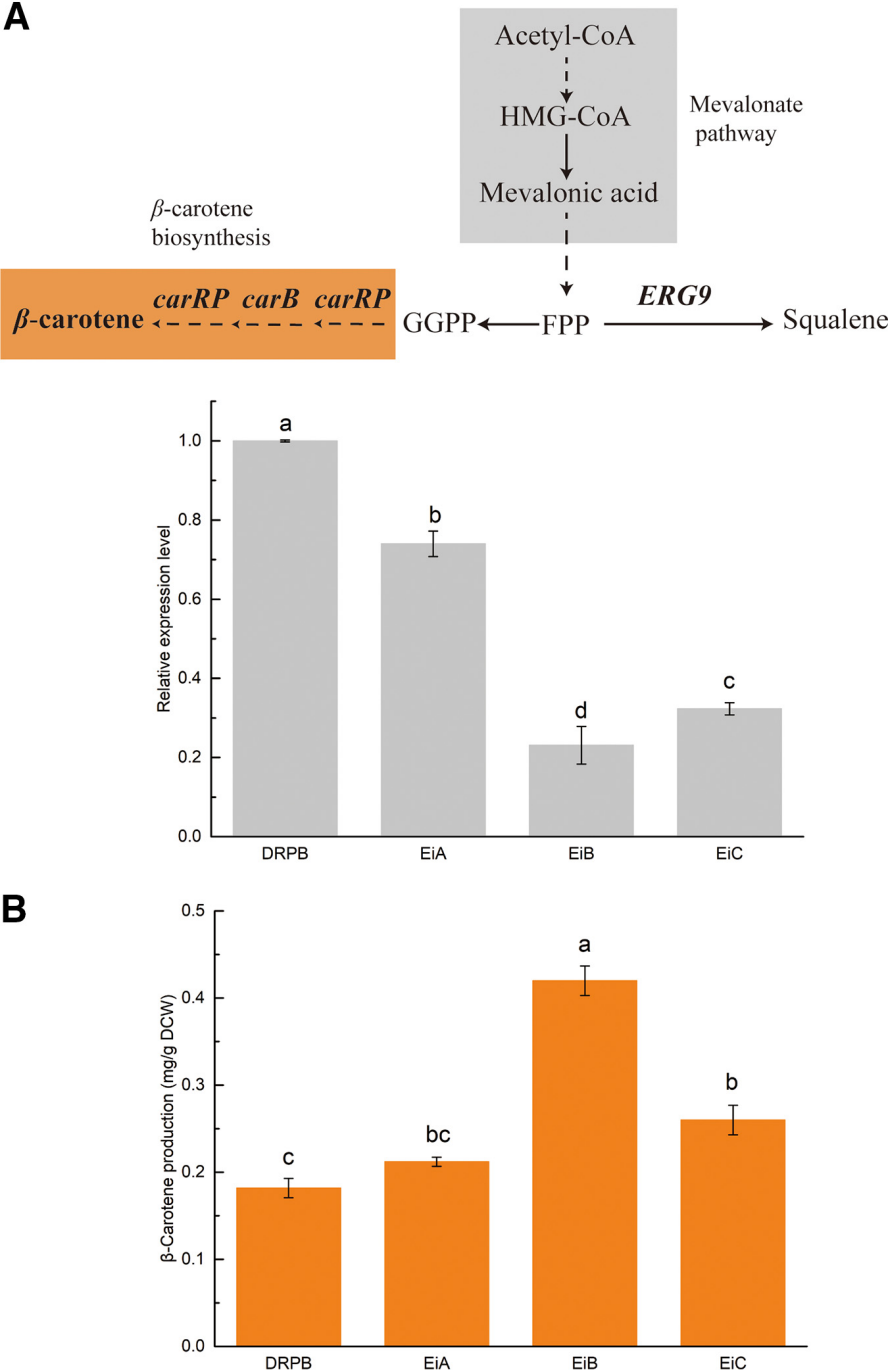
Regulation of β-carotene biosynthesis. (A) The critical pathway of β-carotene biosynthesis in reconstructive C. tropicalis and relative transcription levels of *ERG9* in the yeasts. (B) Yields of β-carotene production in the regulated yeasts. Data are represented as means ± standard deviations from at least three independent occasions. Significant differences are marked with different letters (*P* < 0.05).

In a previous study, a heterologous β-carotene synthetic pathway was constructed in C. tropicalis DRPB (32). In the metabolic pathway, GGPP (geranylgeranyl diphosphate) is a common precursor of diterpenoids, and its accumulation reflects terpene production. The sterol biosynthesis pathway is the major farnesyl pyrophosphate (FPP)-consuming pathway in S. cerevisiae, and knockdown of squalene synthase-encoding gene *ERG9* increased production of carotenoid (33). In C. tropicalis DRPB, we aimed to reduce the transcription level of *ERG9* using the CRISPRi system, in order to explore the influence of *ERG9* on β-carotene production (Fig. 7A). Based on valid targeting regions of the genes *GFP3* and *ADE2*, we selected three targets on *ERG9*: EiA (+20), EiB (−41), and EiC (+130). The expression cassette was integrated at the *CAT* locus (Fig. S5). The strain C. tropicalis DRPB was chosen as the control. From the qRT-PCR results, the relative transcription levels of *ERG9* were 74.1 ± 3.2%, 23.1 ± 4.7%, and 32.3% ± 1.5%, in C. tropicalis EiA, C. tropicalis EiB, and C. tropicalis EiC, respectively (Fig. 7A). Next, we analyzed the β-carotene production ability of each strain using high-performance liquid chromatography (HPLC). As shown in Fig. 7B, β-carotene reached 0.42 ± 0.02 mg/g of dry cell weight in C. tropicalis EiB. In C. tropicalis EiA and C. tropicalis EiC, β-carotene reached was 0.21 ± 0.05 and 0.26 ± 0.02 mg/g of dry cell weight, respectively. Comparing the yield of β-carotene with the relative transcription levels, we found that the strain with the lowest expression of *ERG9* accumulated the highest yield of β-carotene. Results in Pearson test showed that the yield of β-carotene and the relative expression level of *ERG9* were negatively related (*r* = −0.820, *P* < 0.05). The knockdown of *ERG9* enhanced the accumulation of β-carotene in C. tropicalis. Additionally, this confirmed that the CRISPRi system could be used for metabolic engineering of C. tropicalis.

## DISCUSSION

In C. tropicalis, the previous CRISPR-Cas9 system employed an RNA Pol II promoter, together with a hammerhead (HH) type ribozyme and a hepatitis D virus (HDV) ribozyme (32). When using the gRNA strategy with the RNA Pol II promoter and HH and HDV ribozymes, secondary structure can be a complication. Additionally, the HH-HDV-based gRNAs did not show high disruption efficiency in C. tropicalis ATCC 20336 compared with that in S. cerevisiae (32). To simplify the construction of gRNAs, we developed a strategy for gRNA expression mediating efficient CRISPR-Cas9-based gene editing and silencing. In the present work, the whole genome sequence of C. tropicalis ATCC 20336 was investigated using bioinformatics methods (30) to identify the coding sequences of various tRNAs. tRNAs with pseudogenes were eliminated since the maturation process of these tRNAs is not clear. Research on the diversity of tRNA genes in eukaryotes shows that tRNA genes in 11 eukaryotes with the same anticodon differ elsewhere in the tRNA structure (34). Indeed, we found high similarity between genes encoding tRNA^Gly^ in C. tropicalis and those in rice and Y. lipolytica (25, 31). Next, we preliminarily selected a tRNA^Gly^ as a candidate for the gRNA expression promoter based on the reported tRNAs in rice (25) and S. cerevisiae (29). The internal elements (box A and B) of tRNAs encoding DNA sequences are believed to support transcription (35), but the promoter-tRNA-gRNA fusion cassette is highly efficient for processing gRNAs (24). Because of this, the endogenous tRNA^Gly^ and the 300-bp sequence upstream of the tRNA^Gly^ were used as the RNA Pol III promoter for constructing a tRNA:gRNA platform in this work. tRNAs have the advantage of short sequences (27), and the tRNA^Gly^ used in this work was only 71 bp. This tiny element is more efficient for rapidly constructing different gRNAs using a simple PCR procedure. Additionally, the tRNA:gRNA platform showed the potential in efficient multi-gene editing in C. tropicalis.

For the created CRISPRi system in this work, *GFP3* and *ADE2* were selected as reporter genes. In the present work, almost identical silencing levels were obtained when targeting within the coding region of *GFP3*, while the silencing levels of *ADE2* fluctuated dependent on the gRNA targeting site within the promoter region. It might be difficult to predict the exact regulation level of targeted genes, as many researchers have different opinions toward the target region for effective gene knockdown, especially in yeasts (15, 36). In S. cerevisiae, 101 gRNA structures on 14 different yeast promoters were tested and a larger number of gRNA-promoter combinations failed in gene knockdown, while some gRNAs enabled nearly 3-fold expression perturbations (15). In the oleaginous yeast Y. lipolytica, 10 gRNAs targeting different regions of *gfp* gene were designed, but no clear correlation between the repression efficiency and targeting sites was found (37).

In metabolic engineering, the flux of a pathway must be tightly regulated to enhance the production of target chemicals (38). In S. cerevisiae, the production of *α*-santalene was improved 2.66-fold with a novel multifunctional CRISPR system (39). In the future, we will further optimize the CRISPRi system by testing different repressors, activators, and other nucleases (e.g., Cas12a) (28). Moreover, other tRNAs will be tested to provide more choices for designing molecular elements. We believe that the developed CRISPRi regulation system could be effectively used in metabolic engineering of C. tropicalis to establish a sophisticated cell factory.

## MATERIALS AND METHODS

### Plasmids, strains, and media.

Plasmids and strains used in this work are listed in Table 2. All plasmids were constructed in Escherichia coli JM109. Media for cultivating C. tropicalis cells were as follows: minimal medium (MM) contained 6.7 g/L yeast nitrogen base, 20 g/L glucose, and 10 g/L (NH_4_)_2_SO_4_, supplemented medium (SM) was supplemented with 0.06 g/L (wt/vol) uracil and based on MM. Fluoroorotic acid SM (FOA-SM) was supplemented with 2 g/L (wt/vol) 5-fluoroorotic acid and based on SM, yeast peptone dextrose (YPD) medium contained 20 g/L (wt/vol) glucose, 20 g/L (wt/vol) peptone, and 10 g/L (wt/vol) yeast extract, and 2× YPD medium contained 40 g/L (wt/vol) glucose, 40 g/L (wt/vol) peptone, and 20 g/L (wt/vol) yeast extract.

**TABLE 2:** Plasmids/strains used in this work

Plasmids/strains	Genotype	Reference
Plasmids		
Ts-*CAT1*-*gda324*-*URA3*	*CAT* gene disruption cassette	Zhang LH et al. (32)
Ts-*CAT2*-*gda324*-*URA3*-*P_GAP1_-GFP3-T_GAP1_*	*GFP3* gene expression cassette, under control of a *GAP1* promoter	Zhang LH et al. (32)
PHCU06	The transient CRISPR-Cas9 cassettes *P_FBA1_*-HH-gRNA3-*URA3*-HDV-*T_FBA1_*-*P_GAP1_*-*Cas9*-*T_ENO1_*^*a*^	Zhang LH et al. (32)
Ts-*CAT2*-*gda324*-*URA3*-P*_GAP1_*-*GFP3*-T*_GAP1_*	*GFP3* gene expression cassette, under control of a *GAP1* promoter	Zhang LH et al. (32)
*ptsgURA3*^*b*^	gRNA expression cassette, *PtRNA^Gly^*-tRNA-gRNA-*URA3*	This work
CPTU	The transient CRISPR-Cas9 cassettes *P_GAP1_-Cas9-T_ENO1_-ptsgURA3*	This work
*ptsgGFP3*^*c*^	gRNA expression cassette	This work
*ptsgGU*^*d*^	gRNA expression cassette	This work
d-*ptsgGU*	*ptsgGU* flanked with the donor of *GFP3* and *URA3*	This work
*ptsgA/B/C/D*	gRNA expression cassette targeting CDS of *GFP3* gene	This work
*ptsgAiA/AiB/AiC/AiD/AiE/AiF/AiG/AiH*	gRNA expression cassette for *ADE2*	This work
*ptsgEiA/EiB/EiC*	gRNA expression cassette for *ERG9*	This work
Ts-*ADE2*-*gda324*-*URA3*	*ADE2* gene disruption cassette	This work
Ts-*POX5-P_GAP1_-GFP3-T_GAP1_-URA3*	*GFP3* gene expression cassette, under control of *GAP1* promoter	This work
Ts-*POX5-P_GAP1_-GFP3-T_GAP1_-URA3-ptsgA*	*GFP3* gene regulation cassette	This work
Ts-*POX5*-*P_GAP1_-GFP3-T_GAP1_-URA3-ptsgB*	*GFP3* gene regulation cassette	This work
Ts-*POX5-P_GAP1_-GFP3-T_GAP1_-URA3-ptsgC*	*GFP3* gene regulation cassette	This work
Ts-*POX5-P_GAP1_-GFP3-T_GAP1_-URA3-ptsgD*	*GFP3* gene regulation cassette	This work
Ts-*POX5*-*gda324*-*URA3*	*POX5* gene disruption cassette	This work
Ts-*POX5-gda324-URA3-ptsgAiA*	*ADE2* gene regulation cassette	This work
Ts-*POX5-gda324-URA3-ptsgAiB*	*ADE2* gene regulation cassette	This work
Ts-*POX5-gda324-URA3*-*ptsgAiC*	*ADE2* gene regulation cassette	This work
Ts-*POX5-gda324-URA3*-*ptsgAiD*	*ADE2* gene regulation cassette	This work
Ts-*POX5-gda324-URA3*-*ptsgAiE*	*ADE2* gene regulation cassette	This work
Ts-*POX5-gda324-URA3*-*ptsgAiF*	*ADE2* gene regulation cassette	This work
Ts-*POX5-gda324-URA3*-*ptsgAiG*	*ADE2* gene regulation cassette	This work
Ts-*POX5-gda324-URA3*-*ptsgAiH*	*ADE2* gene regulation cassette	This work
Ts-*CAT*-*gda324*-*URA3*-*ptsgEiA*-*P_GAP1_*-*dSpCas9*-*T_ENO1_*-*CAT*	*ERG9* gene regulation cassette	This work
Ts-*CAT*-*gda324*-*URA3*-*ptsgEiB*-*P_GAP1_*-*dSpCas9*-*T_ENO1_*-*CAT*	*ERG9* gene regulation cassette	This work
Ts-*CAT*-*gda324*-*URA3*-*ptsgEiC*-*P_GAP1_*-*dSpCas9*-*T_ENO1_*-*CAT*	*ERG9* gene regulation cassette	This work
Strains		
C. tropicalis ATCC 20336	Wild type	ATCC
C. tropicalis Cu-206	*ura3/ura3*	Zhang LH et al. (32)
C. tropicalis Cu-1	*ura3/ura3 CAT/cat::SpCas9*	This work
C. tropicalis GU	*ura3/ura3 CAT/cat::SpCas9 POX5*/*pox5::P_GAP1_-GFP3-T_GAP1_-URA3*	This work
C. tropicalis D1	*ura3/ura3 CAT/cat::dSpCas9*	This work
C. tropicalis Da	*ura3/ura3 ADE2*/*ade2 CAT/cat::dSpCas9*	This work
C. tropicalis Gi0	*ura3/ura3 CAT/cat:: dSpCas9 POX5*/*pox5::P_GAP1_-GFP3-T_GAP1_-URA3*	This work
C. tropicalis GiA	*ura3/ura3 CAT/cat:: dSpCas9 POX5*/*pox5::P_GAP1_-GFP3-T_GAP1_-URA3-ptsgA*	This work
C. tropicalis GiB	*ura3/ura3 CAT/cat:: dSpCas9 POX5*/*pox5::P_GAP1_-GFP3-T_GAP1_-URA3-ptsgB*	This work
C. tropicalis GiC	*ura3/ura3 CAT/cat:: dSpCas9 POX5*/*pox5::P_GAP1_-GFP3-T_GAP1_-URA3-ptsgC*	This work
C. tropicalis GiD	*ura3/ura3 CAT/cat:: dSpCas9 POX5*/*pox5::P_GAP1_-GFP3-T_GAP1_-URA3-ptsgD*	This work
C. tropicalis Ai1	*ura3/ura3 ADE2/ade2 CAT/cat:: dSpCas9 POX5*/*pox5::gda324-URA3*	This work
C. tropicalis AiA	*ura3/ura3* ADE2/*ade2 CAT/cat:: dSpCas9 POX5*/*pox5::gda324-URA3-ptsgAiA*	This work
C. tropicalis AiB	*ura3/ura3* ADE2/*ade2 CAT/cat:: dSpCas9 POX5*/*pox5::gda324-URA3-ptsgAiB*	This work
C. tropicalis AiC	*ura3/ura3* ADE2/*ade2 CAT/cat:: dSpCas9 POX5*/*pox5::gda324-URA3-ptsgAiC*	This work
C. tropicalis AiD	*ura3/ura3* ADE2/*ade2 CAT/cat:: dSpCas9 POX5*/*pox5::gda324-URA3-ptsgAiD*	This work
C. tropicalis AiE	*ura3/ura3* ADE2/*ade2 CAT/cat:: dSpCas9 POX5*/*pox5::gda324-URA3-ptsgAiE*	This work
C. tropicalis AiF	*ura3/ura3 ADE2*/*ade2 CAT/cat:: dSpCas9 POX5*/*pox5::gda324-URA3-ptsgAiF*	This work
C. tropicalis AiG	*ura3/ura3 ADE2*/*ade2 CAT/cat:: dSpCas9 POX5*/*pox5::gda324-URA3-ptsgAiG*	This work
C. tropicalis AiH	*ura3/ura3* ADE2/*ade2 CAT/cat:: dSpCas9 POX5*/*pox5::gda324-URA3-ptsgAiH*	This work
C. tropicalis DRPB	*ura3::carB-carRP/ura3::carB-carRP CAT/CAT*	Zhang LH et al. (32)
C. tropicalis EiA	*ura3::carB-carRP/ura3::carB-carRP CAT*/*cat*::*gda324*-*URA3*-*ptsgEiA*-*P_GAP1_*-*dSpCas9*-*T_ENO1_*	This work
C. tropicalis EiB	*ura3::carB-carRP/ura3::carB-carRP CAT*/*cat*::*gda324*-*URA3*-*ptsgEiB*-*P_GAP1_*-*dSpCas9*-*T_ENO1_*	This work
C. tropicalis EiC	*ura3::carB-carRP/ura3::carB-carRP CAT*/*cat*::*gda324*-*URA3*-*ptsgEiC*-*P_GAP1_*-*dSpCas9*-*T_ENO1_*	This work

a *HH*, a hammerhead type ribozyme-encoding gene; HDV, a hepatitis D virus ribozyme-encoding gene.

b *ptsgURA3*, the plasmid contains the tRNA:gRNA array targeting *URA3*.

c *ptsgURA3*, the plasmid contains the tRNA:gRNA array targeting *GFP3*.

d *ptsgGU*, the plasmid contains the tRNA:gRNA array targeting *GFP3* and *URA3*.

### Analysis of endogenous tRNAs based on the genome sequence.

tRNAscan-SE (30) was used for searching and subsequent analysis of tRNAs in C. tropicalis ATCC 20336. Genome information was obtained from our previous study (32). The endogenous tRNA^Gly^ and the 300-bp upstream sequence were obtained as the RNA Pol III promoter for producing gRNAs and were applied in both CRISPR-Cas9 and the CRISPRi system in this work.

### Construction and verification of the gRNA expression cassette.

The tRNA^Gly^ and the 300-bp sequence upstream of tRNA^Gly^ were amplified from the genome of C. tropicalis ATCC 20336. The tRNA:gRNA platform targeting *URA3*, *ptsgURA3*, was chemically synthesized (Genewiz, Suzhou, China). Other gRNA expression plasmids were derived from the plasmid *ptsgURA3* by PCR.

For the single-gene disruption, the Cas9 expression cassette was obtained by digesting plasmid PHCU06 (32) with restriction enzyme SpeI. The *ptsgURA3* fragment was obtained by digesting plasmid *ptsgURA3* with restriction enzyme SpeI. *Cas9-ptsgURA3* was constructed by ligation of the above two fragments using DNA ligation kit (TaKaRa, Dalian, China). For the multi-gene disruption, plasmid *ptsgGU* and the tRNA:gRNA platform were constructed from the plasmids *ptsgGFP3* and *ptsgURA3* using a one-step cloning kit (Vazyme, Nanjing, China). The selection of gRNAs and the construction of donors were performed according to our previous study (40). N20 sequences were selected by the bioinformatics tool sgRNACas9 (32, 40).

Verification of the tRNA^Gly^ availability was performed using the transient *Cas9* expression cassette fused with the *ptsgURA3* fragment. The final *Cas9-ptsgURA3* construct and the donor were transformed into C. tropicalis ATCC 20336. The donors contained a stop codon and 50-bp homologous regions upstream and downstream of the cleavage sites in *URA3* (orotidine monophosphate decarboxylase) and *GFP3*, respectively. The donor of *URA3* was inserted into the EcoRI restriction enzyme, and the donor of *GFP3* was inserted into the HindIII restriction enzyme site. The donor was obtained by PCR using each pair of equimolar diluted DNA primers. Primers for the donor of *URA3* and *GFP3* for homology-directed repair (HDR) are listed in Table S2. To explore the functions of the tRNA:gRNA platform, the fragment of the array flanked with the donors was transformed into C. tropicalis GU, which was integrated with *GFP3* and *URA3* at the *POX5* (acyl coenzyme A oxidase I) locus. The editing rates were calculated from three independent experiments.

### Construction of the CRISPRi system in C. tropicalis.

With the aid of tRNAs, we constructed the CRISPRi system by choosing C. tropicalis Cu-206 as the chassis cell and integrated *dSpCas9* at the *CAT* locus. We further expressed *GFP3* at the *POX5* locus to assess the efficiency of the CRISPRi system in C. tropicalis D1. In the control stain C. tropicalis Gi0, *GFP3* was expressed utilizing a *GAP1* promoter, while gRNA expression cassettes fused to the terminator of *GFP3* were expressed in C. tropicalis GiA, GiB, GiC, and GiD. For assessing *ADE2* regulation efficiency, the construction steps for gRNA expression cassettes were similar, except that a copy of *ADE2* was knocked out in C. tropicalis D1. All expression cassettes were flanked with the MluI restriction enzyme for digestion. DNA fragments were purified by the ethanol precipitation method. Yeast transformation employed the lithium chloride protocol, and the selection marker *URA3* was recycled after each transformation (41). C. tropicalis is a diploid yeast, and all expression cassettes were integrated at a single locus, which was verified by PCR and DNA sequencing analysis.

### Fluorescence measurement of *GFP3*.

Recombinant strains expressing *GFP3* were cultivated until reaching an OD_600_ of 10 in 20 mL YPD medium and then inoculated into fresh synthetic medium with an initial OD_600_ of 0.05 in 50 mL YPD. Cells at a final OD_600_ of 2 to 3 were pelleted by centrifugation, washed twice with phosphate-buffered saline (PBS), and resuspended in PBS at an optimal density. Fluorescence images were obtained by a Nikon Eclipse 80i microscope (Nikon Corp., Tokyo, Japan) equipped with a blue laser (488 nm excitation, 530 nm emission).

### Mean fluorescence intensity measurement of *GFP3*.

Recombinant strains expressing *GFP3* were cultivated until reaching an OD_600_ of 10 in 20 mL YPD medium and then inoculated into fresh synthetic medium with an initial OD of 0.05 in 50 mL YPD. Cells at a final OD_600_ of 2 to 3 were pelleted by centrifugation, washed twice with phosphate-buffered saline (PBS), and resuspended in PBS at an optimal density. Mean fluorescence intensity (MFI) was measured by a BD FACSAria III instrument (Becton, Dickinson and Company, Shanghai, China) with a blue laser (488 nm excitation, 530 nm emission).

### qRT-PCR.

C. tropicalis cells at an OD_600_ of 1.5 to 2.5 were collected from the YPD fermentation broth, and total RNAs were isolated using a yeast RNAiso kit (TaKaRa, Dalian, China). RNA samples were further transcribed into cDNA with a PrimeScript RT reagent kit and gDNA eraser (TaKaRa, Dalian, China) following the manufacturer’s instructions. To determine the relative expression levels via quantitative PCR (qPCR), the housekeeping gene *ACT1* (accession no. FM864204.1) was used as a control. All qPCR experiments were carried out with a SYBR premix *Ex Taq* kit (TaKaRa, Dalian, China) and a CFX96 real-time PCR system (Bio-Rad, CA, USA). Primers for *ACT1*, *GFP3*, and *ADE2* were CTTACGAATTGCCAGATG (qACT1-F), CTTACGAATTGCCAGATG (qACT1-R), GACACAACATTGAAGATGGT (qGFP3-F), GCAGATTGAGTGGATAAGTAAT (qGFP3-R), CCAGAATCTTGAAGCAGTT (qADE2-F), and CAGCACCAGCAATGATAC (qADE2-R).

### Extracting β-carotene.

Recombinant strains accumulating β-carotene were cultivated to an OD_600_ of 15 to 20 in 15 mL YPD medium and then inoculated into fresh synthetic medium to an initial OD of 0.1 in 15 mL YPD. After being cultivated for 96 h, two 1.5-mL samples of fermentation broth were collected by centrifugation and washed twice using double-distilled water (ddH_2_O). The cellular precipitate from one sample was dried for measuring dry cell weight. The second sample was mixed with 1 mL 3 M HCl and boiled for 3 min, and the cell precipitate was collected by centrifugation (8,000 rpm) and washed with ddH2O. After being mixed with 1 mL acetone, cells were collected by centrifugation for 10 min (8,000 rpm). The supernatant liquid served as β-carotene extracting solution and was subjected to high-performance liquid chromatography (HPLC) analysis. Concentrations of β-carotene in culture broths were analyzed by Agilent 1260 Infinity (Agilent Technologies, Wilmington, USA) coupled with UV detection. Mobile phases A, B, and C were 35% methanol, 35% acetonitrile, and 30% ethyl acetate in water, respectively. The flow rate of the mobile phases was 1 mL/min, and the column temperature was 25°C. UV wavelength was 450 nm.
