# Supplementary material for: Development of a gRNA Expression and Processing Platform for Efficient CRISPR-Cas9-Based Gene Editing and Gene Silencing in Candida tropicalis
Source: Microbiol Spectr. 2022 May 11;10(3):e00059-22. doi: 10.1128/spectrum.00059-22 (PMC9241840; doi:10.1128/spectrum.00059-22)
Supplement: SUPPLEMENTAL FILE 1 — Supplemental material. Download spectrum.00059-22-s001.pdf, PDF file, 1 MB [file spectrum.00059-22-s001.pdf]

Supporting Information for

Development of a gRNA expression and processing platform for efficient CRISPR-Cas9-based gene editing and gene silencing in *Candida tropicalis*

Yujie Li[1,2], Lihua Zhang[1,2], Haiquan Yang[1,2], Yuanyuan Xia[1,2], Liming Liu[1,3], Xianzhong Chen[1,2,*], Wei Shen[1,2,*]

[1]Key Laboratory of Industrial Biotechnology, Ministry of Education, Jiangnan University,Wuxi, China

[2]School of Biotechnology, Jiangnan University, Wuxi, China

[3]State Key Laboratory of Food Science and Technology, Jiangnan University, Wuxi, China

[*] **Correspondence**

Xianzhong Chen

Mailing address: Key Laboratory of Industrial Biotechnology, Ministry of Education, Jiangnan University, 214122 Wuxi, China.

Email: xzchen@jiangnan.edu.cn

Wei Shen

Mailing address: Key Laboratory of Industrial Biotechnology, Ministry of Education, Jiangnan University, 214122 Wuxi, China.

Email: shenwei@jiangnan.edu.cn

Table of Contents:

Fig. S1 The alignment of endogenous $tRNA^{Gly}$ sequences in *C. tropicalis* ATCC 20336, *C. tropicalis* MYA 3404 and *C. tropicalis* 121.

Fig. S2 Multi-gene disruption with the tRNA-gRNA array.

Fig. S3 The growth curve of *C. tropicalis* in SM medium.

Fig. S4 Downregulation of *ADE2* in *C. tropicalis.*

Fig. S5 *ERG9* gene regulation cassette.

Table. S1 The sequences of N20 in sgRNAs.

Table. S2 Primers in gene deletion experiments.

Sequence. S1 *ptsgURA3* DNA sequence.

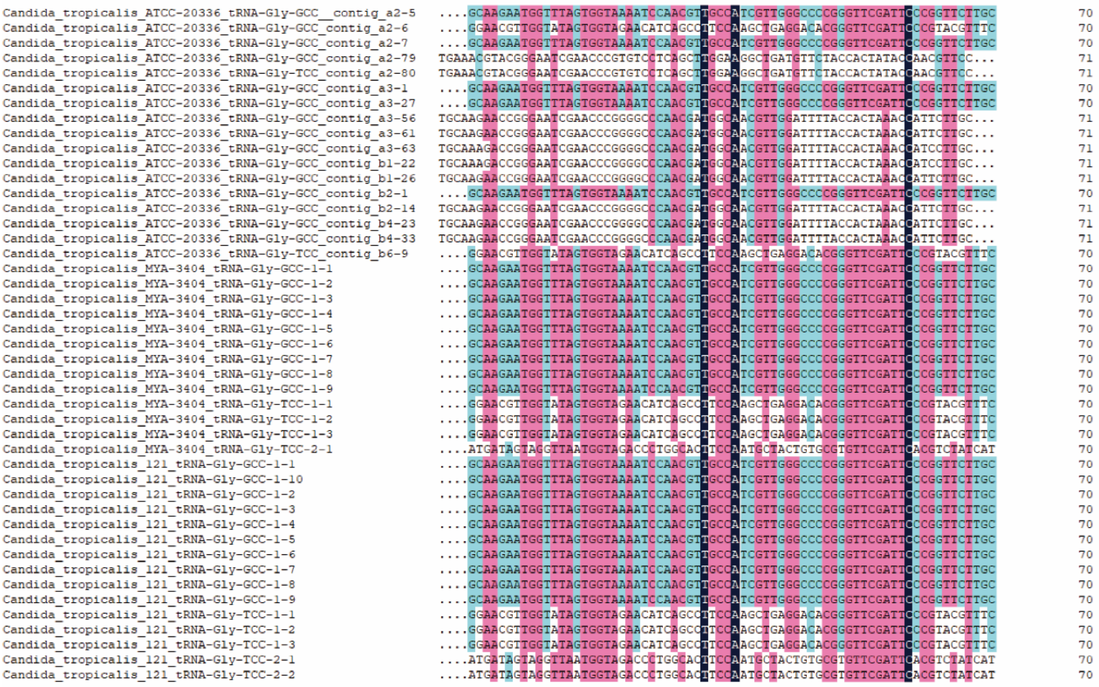

Fig. S1 The alignment of endogenous $tRNA^{Gly}$ sequences in *C. tropicalis* ATCC 20336, *C. tropicalis* MYA 3404 and *C. tropicalis* 121.

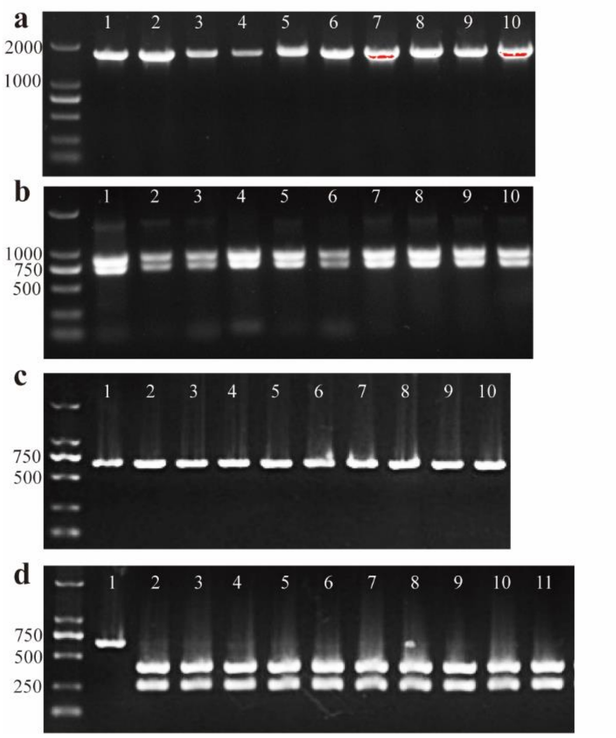

Fig. S2 Multi-gene disruption with the tRNA-gRNA array. **a**: PCR products of *ura3* gene (1.6 kb). **b**: *Eco*RI digested PCR products of *ura3* gene. Lane 1-10 (0.7 kb, 0.9 kb) represents that the restriction enzyme site *Eco*RI was integrated into the genome. **c**: PCR products of *gfp3* gene (0.7 kb). **d**: *Hin*dIII digested PCR products of *gfp3* gene. Lane 1 represents the failed editing, as the restriction enzyme site *Hin*dIII was not integrated into the genome. Lane 2-11 (0.3 kb, 0.4 kb) shows the successful editing. The PCR templates were genome DNA of individual clones randomly picked from FOA-SM plates. FOA-SM plate, SM supplemented with 2 g/L (w/v) 5-fluoroorotic acid.

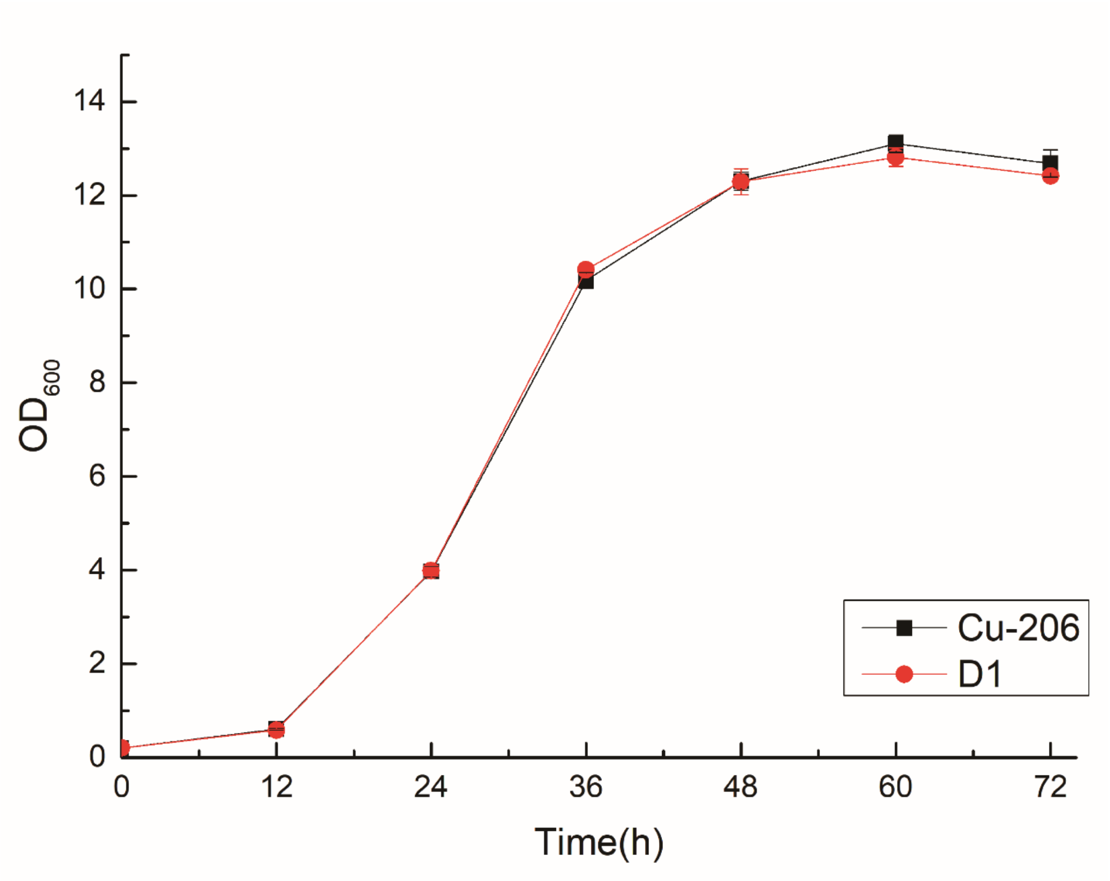

Fig. S3 The growth curve of *C. tropicalis* in SM medium. The data represents the mean ± standard deviations of biological triplicates.

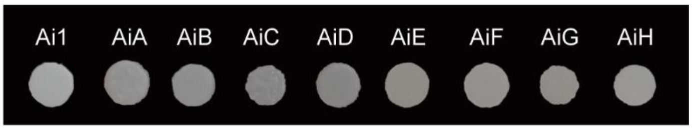

Fig. S4 Downregulation of *ADE2* in *C. tropicalis*. Colony phenotypes of the mutants on MM plates. The names of the strains were labelled in the abbreviation form (e.g., Ai1 refers to *C. tropicalis* Ai1).

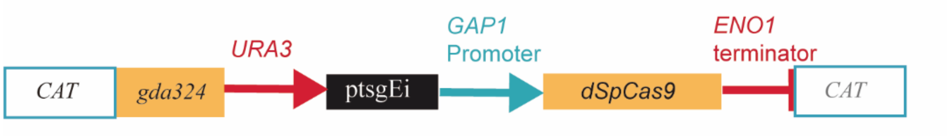

Fig. S5 *ERG9* gene regulation cassette. *ptsgEi* was designed for targeting *ERG9*.

Table. S1 The sequences of N20 in sgRNAs.

GFP3	GiA	AAAGGTGAAGAATTATTCAC
	GiB	GAATTAGATGGTGATGTTAA
	GiC	GGTGAAGGTGATGCTACTTA
	GiD	TTCAAGTCTGCCATGCCAGA
ADE2	AiA	CTACGAAAGTATCGTCAAGT
	AiB	GACTCCTCCCTTTAAGATGG
	AiC	TGTCACGTGGATCACTCACG
	AiD	AATGGATATATTTCAATGTT
	AiE	TCTATGTGTGTGTGATGAGA
	AiF	TAATAACCACCAATTAACTC
	AiG	TGAGTGATCCACGTGACACA
	AiH	AGCTTAATCAGACGAAGAAT
ERG9	EiA	ATTAGCTCACCCAACCGAGT
	EiB	TCCCTTTGCCCCCTCCATCT
	EiC	AGTTGTTGAACTTGACTTCT

Table. S2 Primers in gene deletion experiments.

Name	Sequence	Note
URA3-F	TACTCTAACGACGGGTACAAC	PCR verification
URA3-R	ACCCGATTTCAAAAGTGCAG	
GFP3-F	AAGAATTATTCACTGGTGTTGTCC	
GFP3-R	CATGGGTAATACCAGCAGCAG	
Ur-F	GTCACTCCACGACGCGATCCGATACACCCCCGAGGTGTACTGCAACATCACTTA**GAATTC**TTTCCGATAT	Donors for HDR repair (The restriction enzyme cutting sites are underlined.)
Ur-R	CGCCACGGGTTCTTGATCTTTGAAGATAGGAAGTTTGCGGATATCGGAAA**GAATTC**TAAGTGATGTTG	
Gr-F	AGATACCCAGATCATATGAAACAACATGACTTTTTCAAGTCTGCCATGCCATAA**AAGCTT**AGAAGGTT	
Gr-R	TGTAGTTACCGTCATCTTTGAAAAAAATAGTTCTTTCTTGAACATAACCTTCT**AAGCTT**TTATGGCATGG	

Sequence. S1 *ptsgURA3* DNA sequence.

ACGCGTACTAGTACCCGTGGAGAGCTCTGTGGCACCACCGCTTTGTTGATC

TGCCTGTTGGTGTGTGTGGGTGTTACTCCTTTTTCTTGGTATTTTCTTGTGG

TTTCTCTTAACAACGTCGGGCTACTCTTGGGCATGCCCTATCAAACTATTG

CGCAACTCCTGATCCGCATACCCAAAGACGTGCCAGGGGAGTTTGAAGTA

TTCCTGGTGTGACGGCGACAGTCCTGATGAACATTCTCCCTTCCATTTGAC

ATCGCCGGGTCTTTCGACACTAGCAAACGTCCAACCAATCGGACACATGT

CTGAGTTGCAAGAATGGTTTAGTGGTAAAATCCAACGTTGCCATCGTTGG

GCCCCGGGTTCGATTCCCGGTTCTTGCACGAGGTGTACTGCAACATCAGTT

TTAGAGCTAGAAATAGCAAGTTAAAATAAGGCTAGTCCGTTATCAACTTG

AAAAAGTGGCACCGAGTCGGTGCTTTTTTTTTTTT***ACGCGT***

The black characters in bold italic indicates the restriction enzyme cutting site

***ACGCGT* (*Mlu*I) *ACTAGT* (*Spe*I)**

The green characters indicate the 300 bp sequence upstream of the $tRNA^{Gly}$

The blue characters indicate the $tRNA^{Gly}$ coding sequence

The black characters underlined indicate gRNA scaffold

The black characters indicate poly(dT)
